# Causal relationship between rheumatoid arthritis and epilepsy in a European population: a univariate and multivariate Mendelian randomization study

**DOI:** 10.3389/fimmu.2024.1389549

**Published:** 2024-05-16

**Authors:** Chang Liu, Jiangnan Ye, Shixiu He, Zhijun Ma, Fang Luo, Jintao Miao, Huinan Li, Puhua Cao, Jun Zhu

**Affiliations:** ^1^ School of Acupuncture-Moxibustion and Tuina, Chengdu University of Traditional Chinese Medicine, Chengdu, Sichuan, China; ^2^ The Third Affiliated Hospital of Chengdu University of Traditional Chinese Medicine, Chengdu, Sichuan, China; ^3^ Arthrology Department, Nanchong Gaoping District People’s Hospital, Nanchong, Sichuan, China; ^4^ School of Clinical Medicine, Chengdu University of Traditional Chinese Medicine, Chengdu, Sichuan, China

**Keywords:** rheumatoid arthritis, epilepsy, Mendelian randomization, autoimmunity, central nervous system

## Abstract

**Background:**

Several previous studies have reported an association between rheumatoid arthritis (RA) and epilepsy, but the causal relationship is unclear. The aim of this study was to assess the connection between RA and epilepsy in a European population using Mendelian randomization (MR).

**Methods:**

Genome-wide association study summary data on RA and epilepsy from European populations were included. Univariate MR (UVMR) and multivariate MR were used to investigate the causal relationship between the two conditions. Three analysis methods were applied: inverse variance weight (IVW), MR-Egger, and weighted median, with IVW being the primary method. Cochran Q statistics, MR-PRESSO, MR-Egger intercept, leave-one-out test, and MR-Steiger test were combined for the sensitivity analysis.

**Results:**

UVMR showed a positive association between RA and epilepsy risk (OR=1.038, 95% CI=1.007–1.038, p=0.017) that was supported by sensitivity analysis. Further MVMR after harmonizing the three covariates of hypertension, alcohol consumption, and smoking, confirmed the causal relationship between RA and epilepsy (OR=1.049, 95% CI=1.011–1.087, p=0.010).

**Conclusion:**

This study demonstrated that RA is associated with an increased risk of epilepsy. It has emphasized that the monitoring of epilepsy risk in patients diagnosed with RA should be strengthened in clinical practice, and further studies are needed in the future to explore the potential mechanism of action connecting the two conditions.

## 1 Introduction

Epilepsy is a prevalent and highly disabling chronic central nervous system (CNS) disorder characterized by sudden abnormal discharges of neurons in the brain, resulting in transient brain dysfunction. It affects more than 70 million people globally (1, 2), with the highest prevalence and incidence in infants and the elderly and slightly higher rates in men than women (3). People with epilepsy have a lower risk of death and shorter life expectancy than the general population (4), and uncontrollable seizures make them highly vulnerable to accidental injuries, such as traffic accidents, drowning, falls, and burns (5). Additionally, epilepsy puts patients at an increased risk for psychiatric disorders and suicide (6, 7), placing a heavy burden on these patients, both psychologically and physically. Although multiple underlying disease mechanisms have been identified as being associated with epilepsy, the specific etiology of approximately 50% of epilepsy cases globally remains incompletely understood (8).

Rheumatoid arthritis (RA) is a chronic rheumatic disease characterized by persistent synovitis, primarily affecting the joints, which can lead to severe bone and cartilage damage and disability (9). The prevalence of RA is estimated to be close to 1% of the total global population (10). Because of its severe impact on quality of life, the need for lifelong treatment to improve symptoms, and the development of multiple complications, it has a tremendous impact on individuals and society (11). In recent years, numerous studies have reported an association between RA and epilepsy (12–20) and shown an increased risk of epilepsy in patients with RA or an increased risk of epilepsy in children of parents with RA, although some studies have provided inconsistent evidence (21). Observational studies are greatly limited in their ability to infer causality due to the influence of difficult-to-control confounding factors and reverse causation. Randomized controlled studies, which are the gold standard for inferring causality, are also difficult to implement because of ethical, economic, and time restraints. At present, we are unsure whether there is a causal relationship between RA and epilepsy.

Mendelian randomization (MR) is an innovative epidemiological approach that uses genetic variants, such as single nucleotide diversity (SNPs), as instrumental variables (IVs) to infer potential causal relationships between exposure and outcomes (22, 23). Genetic variants are formed at conception through random assignment, which means they are not affected by other factors, such as behavioral, environmental, and social influences. Therefore, MR avoids the influence of confounding factors and reverse causation to the greatest extent possible (24). A genome-wide association study (GWAS) can provide us with data on the IVs associated with specific types of exposure, allowing us to use GWAS findings for MR analysis (23).

## 2 Materials and methods

### 2.1 Study design

**Figure 1:**
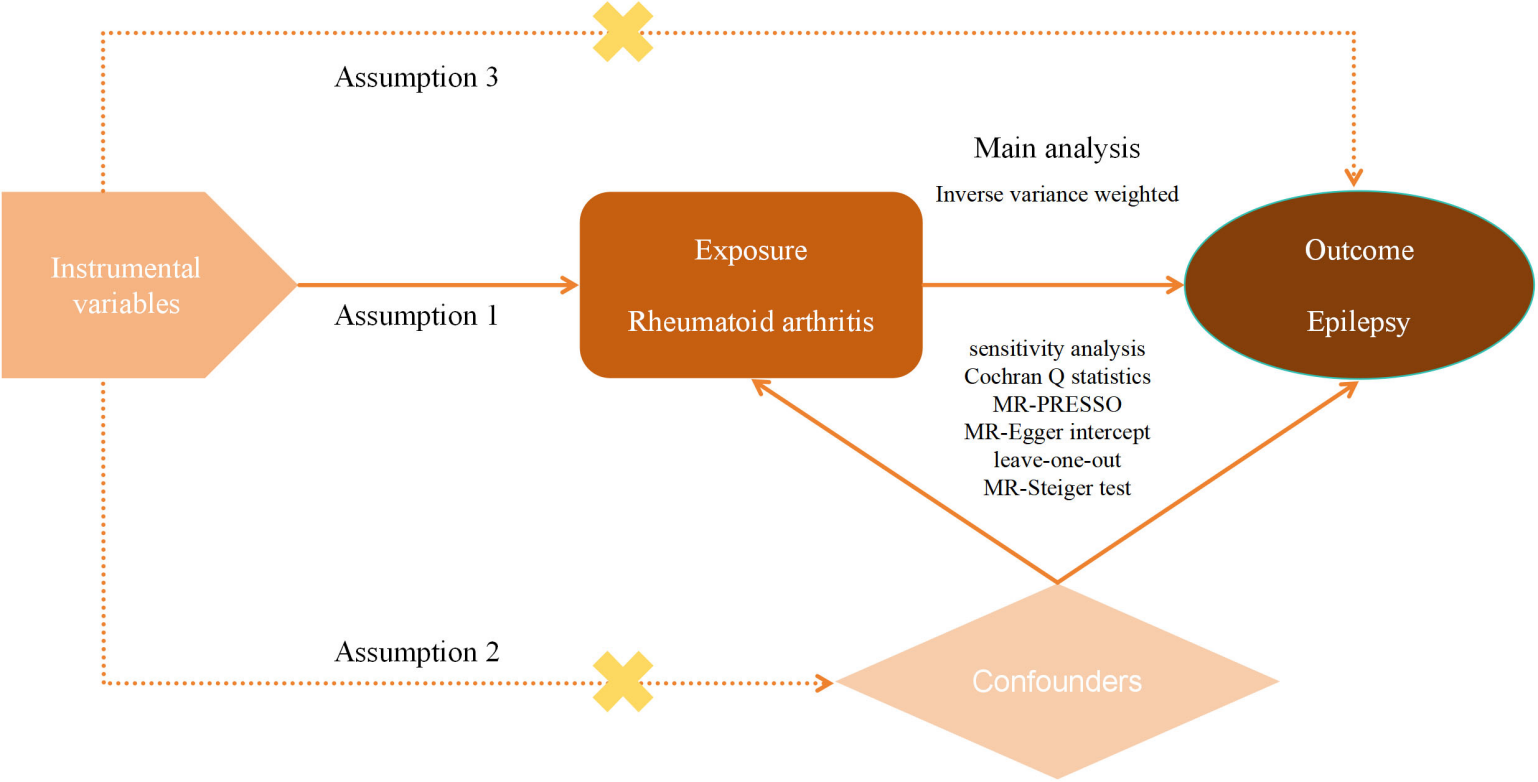
Flowchart of MR study design.

Herein, we included GWAS summary data on epilepsy and RA from a European population. First, classical UVMR was used to verify whether there was a causal relationship between RA and epilepsy. Then MVMR was used to further validate the reliability of this relationship. The purpose of MVMR is to explore the influence of multiple factors on a particular outcome, which helps us determine whether the causal link between the exposure factor of interest and the outcome is affected by other confounders and thus assess the causal effect more precisely. To ensure the reliability of the results of MR analyses, the selected IVs had to fulfill three key assumptions: (1) there was a strong association between the IVs and RA; (2) the IVs were not associated with any other potential confounders that may affect RA and epilepsy; and (3) the IVs affect epilepsy only through RA. Moreover, the design of this study followed the most recent MR guidelines (STROBE-MR) (24). An overview of our study design can be seen in **Figure 1**.

### 2.2 Data sources

GWAS summary data on RA and epilepsy were extracted from the IEU Open GWAS project (https://gwas.mrcieu.ac.uk/). As the data are publicly released and available, no additional ethical approval was required. To entirely avoid the effects of population stratification, our data selection focused only on patients of European ancestry, while samples from other ancestries and mixed ancestries were excluded. In addition, to minimize sample overlap, we chose data from different leagues. The GWAS summary data for RA (GWAS ID: ebi-a-GCST90013534) were derived from a GWAS meta-analysis of 58,284 mixed-sex individuals, including 14,361 cases and 43,923 controls, with 13,108,512 SNPs. Patients with RA were diagnosed by rheumatologists or according to the 1987 American College of Rheumatology criteria (25). The GWAS summary data for epilepsy (GWAS ID: finn-b-G6_EPLEPSY) were obtained from the FinnGen Consortium, and all information on these data can be found in Risteys FinnGen R12 (https://risteys.finregistry.fi/) by searching for G6_ EPLEPSY. The data cover a total of 182,367 individuals of mixed sexes, including 6,260 cases and 176,107 controls, with 16,380,349 SNPs. Epilepsy was diagnosed by epilepsy specialists on the basis of electroencephalogram, magnetic resonance imaging, and clinical history analysis (26). The GWAS summary data for both epilepsy and RA were selected from the largest sample size datasets originating from individuals of purely European ancestry that were publicly available.

### 2.3 Instrumental variables selection

To fulfill the three key assumptions of MR for selecting IVs, we set up the following stringent criteria to screen SNPs for use as IVs in this study: (1) to ensure that SNPs were significantly associated with our exposure factors of interest, SNPs with P < 5 × 10^−8^ were selected; (2) to avoid the influence of linkage disequilibrium (LD) among selected SNPs, we collected European population samples and used thresholds such as r^2^ < 0.001 and distance = 10,000 kb for LD-clumping to exclude SNPs with strong LD (27, 28); (3) F-statistic was incorporated to ensure that the selected IVs were strong IVs. Variables with F-statistic > 10 were usually defined as strong IVs, and those whose strength did not meet the standard SNPs were excluded, with the formula for F-statistic as follows: F= beta^2^/se^2^; (4) SNPs were manually screened in Phenoscanner (www.phenoscanner.medschl.cam.ac.uk) to ensure they met the above criteria and that they were unaffected by potential confounding factors; (5) Palindromic SNPs with symmetry were eliminated from the MR analysis.

### 2.4 MR analysis

We performed all MR and correlation analyses in R (4.3.2) software using the three R packages TwoSample MR, MR-PRESSOR, and Mendelian randomization. UVMR was first used to investigate whether there was a causal relationship between RA and epilepsy. Then, we used MVMR to further verify the reliability of the previously derived causal relationship after adjusting for hypertension (29), alcohol consumption (30), and smoking (31), three common risk factors for epilepsy reported in previous studies. **Supplementary Table 1** lists the covariates used in the MVMR.

Inverse Variance Weight (IVW), MR-Egger, and weighted median (WM) analyses were used in our study. These methods make different assumptions about possible horizontal pleiotropy: IVW assumes that all IVs are free of horizontal pleiotropy (32); MR-Egger assumes that all IVs are horizontally polytropic (33); and WM assumes that horizontal pleiotropy can exist in 50% of the IVs (34). Of these, IVW served as our primary analytic method because it allows for the most accurate causal assessment in the absence of horizontal pleiotropy (32). If horizontal pleiotropy occurred between SNPs, the results obtained by the two methods MR-Egger and WM were referred to.

### 2.5 Sensitivity analysis

Sensitivity analysis was applied to ensure the robustness of the MR analysis results, and it mainly included heterogeneity analysis, horizontal pleiotropy analysis, the leave-one-out test, and the MR-Steiger test, with the following workflow (1) Cochran Q statistics was used to identify whether the effects between different SNPs were heterogeneous (35), and if heterogeneity existed, MR-PRESSO was employed to exclude the heterogeneity of the larger SNPs (36), and MR analysis was repeated; (2) using MR-Egger regression, we determined whether the SNPs had horizontal pleiotropy based on MR-Egger intercept (33); (3) using the leave-one-out test, we could exclude individual SNPs one by one and reperform the computational MR analysis to assess whether individual SNPs had an impact on the overall study results; (4) The MR-Steiger test was used to verify the correctness of the directionality of the causal effects derived from the study and to avoid interference from reverse causality.

## 3 Results

### 3.1 Instrumental variables

After rigorous screening of IVs according to previously developed criteria, a total of 90 SNPs met the requirements to be included in this study. The SNP rs34536443 was excluded from MR analysis because it was found to have a palindrome with an intermediate allele frequency. All SNPs were strong IVs (F-statistic > 10). Details of the SNPs included in the study can be seen in **Supplementary Table 2**.

### 3.2 UVMR

The IVW method showed a positive association between RA and epilepsy risk (OR=1.038, 95% CI=1.007–1.038, p=0.017); MR-Egger: OR=1.045, 95% CI=0.998–1.095, p=0.066; and WM: OR=0.997, 95%CI=0.949–1.047, p=0.897.

**Figure 2:**
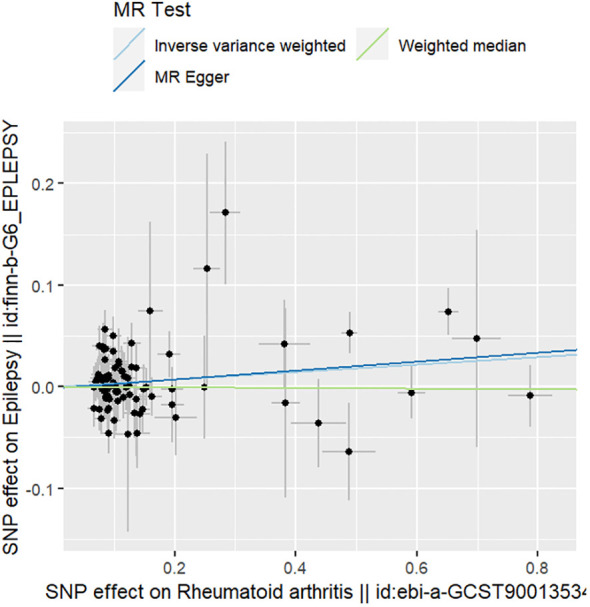
Scatterplot of MR estimates for RA associated with epilepsy.

**Figure 3:**
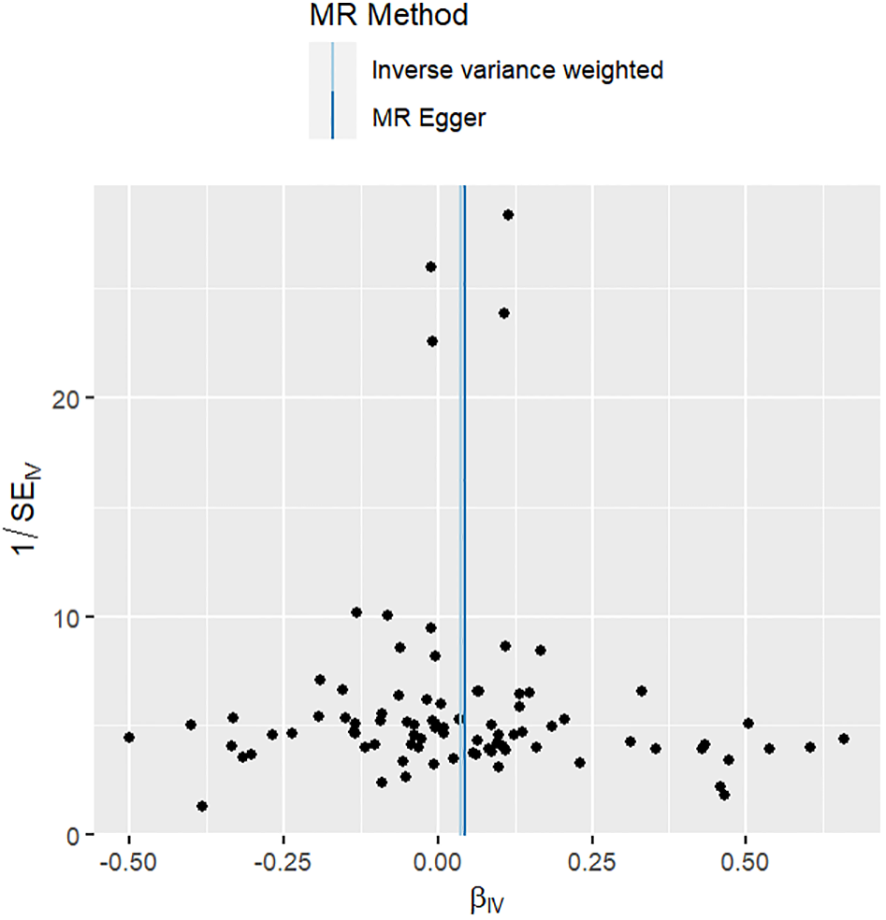
Funnel plots of MR estimates for RA associated with epilepsy.

**Figure 4:**
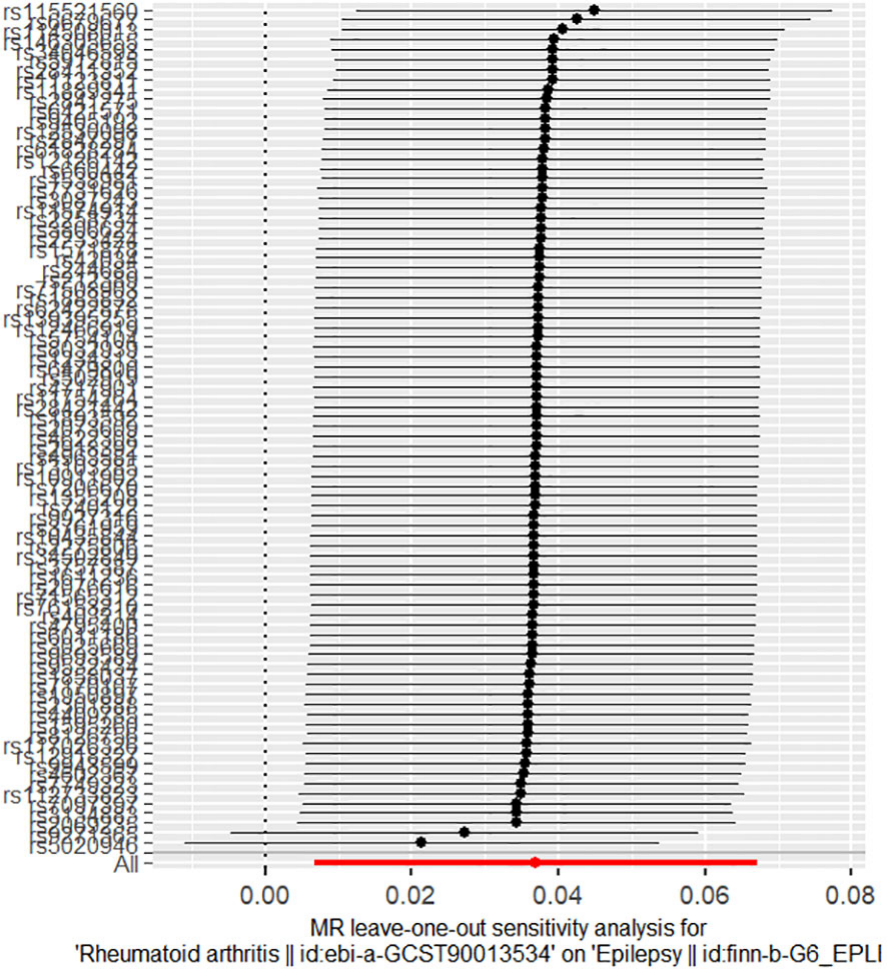
Leave-one-out test plots of MR estimates for RA associated with epilepsy.

The results of the sensitivity analyses supported the hypothesis of a causal relationship between genetically predicted RA and epilepsy. A p-value of >0.05 in the Cochran Q statistics indicated the absence of heterogeneity. A p-value of >0.05 in the MR-Egger intercept test indicated the absence of horizontal pleiotropy. The leave-one-out test indicated that the overall findings were not influenced by any single SNP. The results of the MR-Steiger analysis verified the validity of our findings. Because of the absence of heterogeneity and horizontal pleiotropy, we considered the results obtained by the IVW method to be reliable. The results of analysis with Cochran Q statistics, MR-Egger intercept, and MR-Steiger tests can be seen in **Supplementary Tables 3–5**. can be seen in **Supplementary Tables 3, 4**. **Figures 2**–**4** show the scatter plots, funnel plots, and leave-one-out test plots of the UVMR results for the relationship between RA and epilepsy.

### 3.3 MVMR

**Figure 5:**
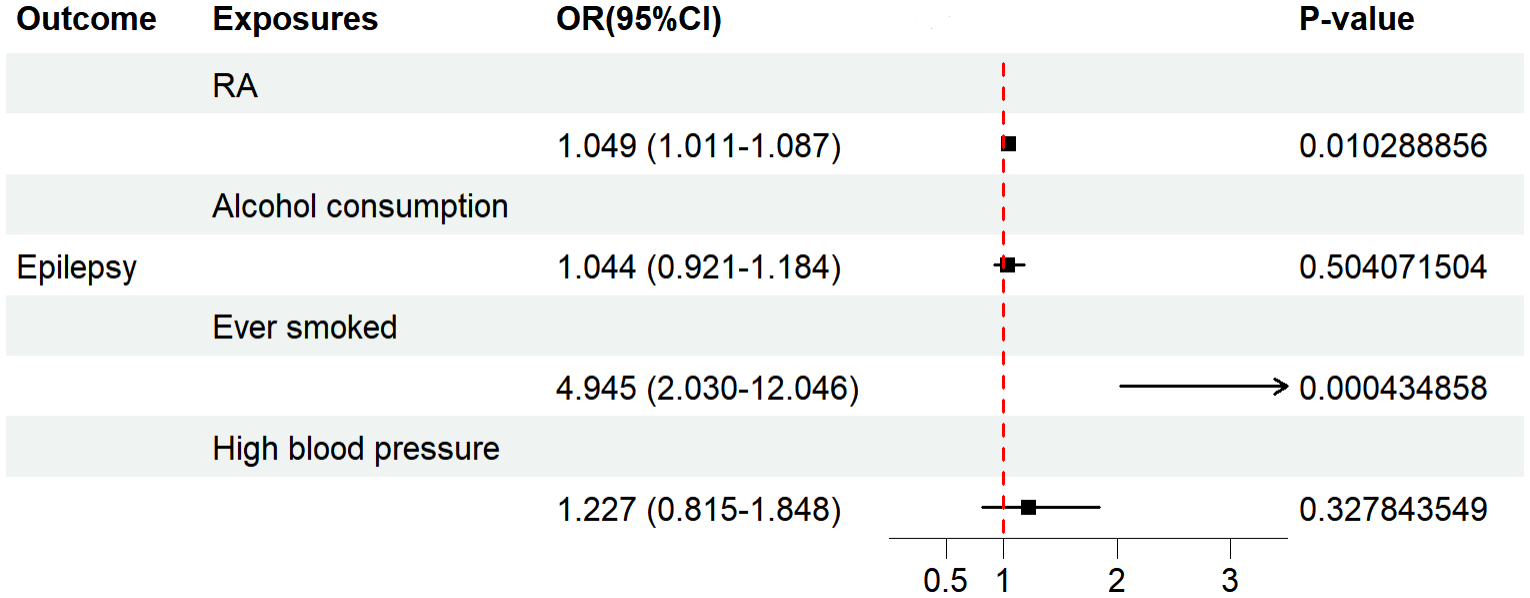
The forest plot depicts the findings of the MVMR analyses on the causal effects of RA on epilepsy. OR, odds ratio; CI, confidence interval; P-value, p value of the causal estimate.

Considering that the results of UVMR may have been affected by potential confounders, we performed further MVMR analysis. After coordinating hypertension, alcohol consumption, and smoking, we used the IVW method to show that there was still a positive causal association between RA and epilepsy risk (OR=1.049, 95% CI=1.011–1.087, p=0.010). **Figure 5** demonstrates the results of the MVMR analysis.

## 4 Discussion

The present study assessed the causal relationship between RA and epilepsy by MR analysis. The results of our UVMR analysis suggest that RA leads to an increased risk of epilepsy, which was confirmed by the results of further MVMR analysis. Our findings provide important clues to further understand the mechanisms underlying this association and emphasize the importance of the timely monitoring and prevention of epilepsy in patients diagnosed with RA.

Our findings are similar to those of several previous large-scale population-based retrospective studies. A study in the United States that included 2,518,034 subjects observed an elevated risk of epilepsy in those with 12 immune disorders, including RA (12). A study by Chang et al. that included 32,005 patients with RA demonstrated a 1.27-fold higher risk of epilepsy in the RA cohort than the control group (32,005 non-RA cases) (14). A study that included 326,415 individuals reported the highest rate of co-morbidity between epilepsy and RA (13). A study including 821 RA cases and 2,455 controls found that RA carried a greater risk of 11 comorbidities, including epilepsy (15). In addition, a bioinformatics study similarly confirmed that RA was strongly associated with epilepsy. Malekpour et al. (16) explored the relationship between frontal lobe epilepsy and three immune disorders, including RA, and showed that there were common genetic variants among the conditions. In addition, a meta-analysis emphasized that patients with RA have a higher risk of epilepsy than non-RA patients, and there was a negative correlation with age. This study also found 13 genes related to inflammatory factors that showed overlapping expression in RA and epilepsy patients (19). However, an early cross-sectional study reported that RA was uncommon in patients with epilepsy (21).

Currently, little is known about the potential factors underlying the theory that RA increases the risk of epilepsy. However, these may include inflammatory responses, autoimmunity, painful stimuli, CNS involvement, and adverse drug reactions. Many studies have shown that inflammation is associated with the development of epilepsy (37–39), and RA is an autoimmune inflammatory disease in which upregulation of the inflammatory cytokines TNF-α, IL-1, and IL-6 is closely related to pathogenesis. Changes in the levels of these inflammatory cytokines have also been observed in epilepsy (40, 41). The persistent and complex inflammatory responses, such as the activation of microglia and astrocytes and production of pro-inflammatory molecules, found in brain tissues of surgically resected patients with refractory epilepsy and in the brain tissues of rat frontal lobe epilepsy models also suggest that the increased risk of epilepsy in RA patients may be associated with the inflammatory component of RA (42, 43). This idea is emphasized by the fact that the use of nonsteroidal anti-inflammatory drugs attenuates seizures or reduces the risk of epilepsy, as reported in several studies (14, 44). On the contrary, there may be an autoimmune component to epilepsy, and some neuronal autoantibodies that may have pathogenic effects have been found in epileptic patients, such as antibodies against the NR1 subunit of the N-methyl D-aspartate receptor (45). High-quality studies have reported that epilepsy patients with these antibodies showed improvements in their symptoms with immunotherapy (46, 47). However, pain, which is the main symptom of RA patients, may also be a possible route to an increased risk of epilepsy. It is well known that the cerebral cortex plays an important role in the pathogenesis of epilepsy. Previous studies have shown that some cerebral cortex structures are involved in pain modulation (48), and studies have also shown that some cortical regions are activated accordingly during pain episodes in RA (49–51). Thus, it is possible that persistent pain stimulation in RA patients leads to the abnormal excitation of certain cortical neurons and thus to the onset of epilepsy. In addition, a number of previous case studies have reported seizures in RA patients due to CNS involvement (52–55). CNS involvement is an extra-articular manifestation of RA that affects the meningitis, cerebral vasculitis, and meningeal rheumatoid nodules. Thick membrane inflammation (54) and CNS involvement with meningeal infiltration may be associated with symptoms such as seizures, which may be another route by which RA leads to an increased risk of epilepsy, although the prevalence of CNS complications in patients with RA has not been investigated at this time. Finally, some medications commonly used in RA, such as corticosteroids, methotrexate, and salazosulfapyridine, also seem to be associated with an increased risk of epilepsy (56–58).

Our study has added support to the hypothesis that a causal relationship exists between RA and epilepsy, which has important clinical significance. First, understanding the causal links may help to develop new therapeutic strategies for epilepsy. The etiology of nearly half of all patients with epilepsy has not yet been elucidated, and at present, clinical treatments for epilepsy mainly focus on seizure control, rarely targeting the underlying etiology. Although medication has produced good results in recent years in patients with epilepsy, drugs are still ineffective in about one-third of patients (59). Such patients with refractory epilepsy can be treated by surgery, but unfortunately, the efficacy of this strategy is also unsatisfactory, with 50% of surgically treated patients experiencing a recurrence of epilepsy within 5 years (60). Therefore, we still need to develop new therapeutic approaches, and exploring the underlying causes of epilepsy is key. Second, the findings may facilitate future studies into the common pathogenesis of the two disorders, which could lead to a better understanding of the nature of these disorders and provide more effective strategies for their future prevention and control. Finally, the findings may help physicians to better prevent epilepsy in clinical practice by following the recommendations regarding the regular neurological assessment and monitoring of patients with RA, and thereby, reduce the incidence or mitigate the effects of epilepsy.

In summary, RA may play an important role in the development of epilepsy. However, the biological mechanisms underlying the associations between the two have not yet been clarified. More studies are needed to further explore these underlying mechanisms and to search for possible common risk factors and therapeutic targets, which will provide more scientific evidence for the prevention and treatment of these two diseases.

To the best of our knowledge, this study is the first to validate the causal relationship between RA and epilepsy using the innovative MR method. MR minimizes the impact of confounding factors and reverse causality and can be performed using existing publicly available and reliable data, making it more cost-effective and feasible than other methods. Additionally, our data sources were all recently recorded GWAS datasets with the largest samples of purely European populations. We performed a series of sensitivity analyses on our findings, all of which ensured the credibility along with the robustness of our findings.

At the same time, we must also recognize some shortcomings. First, the current study was based on patients with European ancestry, so generalizing the results of this study to other populations requires caution. Second, some potential confounders still may have influenced our assessment, as RA and epilepsy have distinct gender and age characteristics, and we were unable to perform stratified analyses because of limited data disclosure. Finally, to the best of our knowledge, there are currently no good methods that can be used to calculate sample overlap between exposure and outcomes. The data we included were all of European origin, with some potential for sample overlap, which may have had some impact on the accuracy of our risk estimates. Therefore, GWAS data that include more details will be needed in the future to assess differences more fully between study populations.

## 5 Conclusions

In conclusion, this study suggests that RA is associated with an increased risk of epilepsy. Based on this finding, we suggest that monitoring epilepsy risk in patients diagnosed with RA, as well as individualized assessments, should be strengthened in clinical practice. Further studies are needed in the future to explore the potential mechanisms of action linking RA and epilepsy.

## Data availability statement

The original contributions presented in the study are included in the article/**Supplementary Material**. Further inquiries can be directed to the corresponding author.

## Author contributions

CL: Conceptualization, Methodology, Software, Writing – original draft, Writing – review & editing. JY: Data curation, Software, Writing – review & editing. SH: Conceptualization, Methodology, Writing – review & editing. ZM: Formal analysis, Software, Writing – review & editing. FL: Data curation, Formal analysis, Writing – review & editing. JM: Visualization, Writing – review & editing. HL: Data curation, Software, Writing – review & editing. PC: Software, Visualization, Writing – review & editing. JZ: Conceptualization, Funding acquisition, Supervision, Writing – review & editing.

## References

[1] Devinsky O Vezzani A O’Brien TJ Jette N Scheffer IE de Curtis M. Epilepsy. Nat Rev Dis Primers. (2018) 4:18024. doi: 10.1038/nrdp.2018.24 29722352

[2] Thijs RD Surges R O’Brien TJ Sander JW. Epilepsy in adults. Lancet. (2019) 393:689–701. doi: 10.1016/S0140-6736(18)32596-0 30686584

[3] Beghi E. The epidemiology of epilepsy. Neuroepidemiology. (2020) 54:185–91. doi: 10.1159/000503831 31852003

[4] Kaiboriboon K Schiltz NK Bakaki PM Lhatoo SD Koroukian SM. Premature mortality in poor health and low income adults with epilepsy. Epilepsia. (2014) 55:1781–8. doi: 10.1111/epi.12789 PMC4232978 25244361

[5] Devinsky O Spruill T Thurman D Friedman D. Recognizing and preventing epilepsy-related mortality: A call for action. Neurology. (2016) 86:779–86. doi: 10.1212/WNL.0000000000002253 PMC4763802 26674330

[6] Keezer MR Sisodiya SM Sander JW. Comorbidities of epilepsy: current concepts and future perspectives. Lancet Neurol. (2016) 15:106–15. doi: 10.1016/S1474-4422(15)00225-2 26549780

[7] Erlangsen A Stenager E Conwell Y Andersen PK Hawton K Benros ME. Association between neurological disorders and death by suicide in Denmark. Jama-Journal Am Med Assoc. (2020) 323:444–54. doi: 10.1001/jama.2019.21834 PMC7042859 32016308

[8] Beghi E Giussani G Abd-Allah F Abdela J Abdelalim A Abraha HN. Global, regional, and national burden of epilepsy, 1990–2016: a systematic analysis for the Global Burden of Disease Study 2016. Lancet Neurol. (2019) 18:357–75. doi: 10.1016/S1474-4422(18)30454-X PMC6416168 30773428

[9] Aletaha D Smolen JS. Diagnosis and management of rheumatoid arthritis A review. Jama-Journal Am Med Assoc. (2018) 320:1360–72. doi: 10.1001/jama.2018.13103 30285183

[10] van der Woude D van der Helm-van Mil AHM. Update on the epidemiology, risk factors, and disease outcomes of rheumatoid arthritis. Best Pract Res Clin Rheumatol. (2018) 32:174–87. doi: 10.1016/j.berh.2018.10.005 30527425

[11] Smolen JS Aletaha D McInnes IB. Rheumatoid arthritis. Lancet. (2016) 388:2023–38. doi: 10.1016/S0140-6736(16)30173-8 27156434

[12] Ong MS Kohane IS Cai T Gorman MP Mandl KD. Population-level evidence for an autoimmune etiology of epilepsy. JAMA Neurol. (2014) 71:569–74. doi: 10.1001/jamaneurol.2014.188 PMC4324719 24687183

[13] Dorrington S Carr E Stevelink SAM Dregan A Woodhead C Das-Munshil J. Multimorbidity and fit note receipt in working-age adults with long-term health conditions. psychol Med. (2022) 52:1156–65. doi: 10.1017/S0033291720002937 32895068

[14] Chang KH Hsu YC Chang MY Lin CL Wu TN Hwang BF. A large-scale study indicates increase in the risk of epilepsy in patients with different risk factors, including rheumatoid arthritis. Med (Baltimore). (2015) 94:e1485. doi: 10.1097/MD.0000000000001485 PMC4616629 26356713

[15] Kronzer VL Crowson CS Sparks JA Myasoedova E Davis JM. Comorbidities as risk factors for rheumatoid arthritis and their accrual after diagnosis. Mayo Clinic Proc. (2019) 94:2488–98. doi: 10.1016/j.mayocp.2019.08.010 PMC6907158 31759675

[16] Malekpour M Salarikia SR Kashkooli M Asadi-Pooya AA. The genetic link between systemic autoimmune disorders and temporal lobe epilepsy: A bioinformatics study. Epilepsia Open. (2023) 8:509–16. doi: 10.1002/epi4.12727 PMC10235559 36929812

[17] Öztürk A Öztürk E Safak AA. Pachymeningitis and epilepsy in rheumatoid arthritis, MRI findings. Rivista Di Neuroradiologia. (2004) 17:187–90. doi: 10.1177/197140090401700208

[18] Rom AL Wu CS Olsen J Jawaheer D Hetland ML Christensen J. Parental rheumatoid arthritis and childhood epilepsy: A nationwide cohort study. Neurology. (2016) 87:2510–6. doi: 10.1212/WNL.0000000000003424 PMC5206999 27856781

[19] Zhao H Li S Xie M Chen R Lu H Wen C. Risk of epilepsy in rheumatoid arthritis: a meta-analysis of population based studies and bioinformatics analysis. Ther Adv Chronic Dis. (2020) 11:2040622319899300. doi: 10.1177/2040622319899300 32095225 PMC7011323

[20] Jølving LR Nielsen J Kesmodel US Nielsen RG Beck-Nielsen SS Nørgård BM. Children born by women with rheumatoid arthritis and increased susceptibility for chronic diseases: A nationwide cohort study. Arthritis Care Res (Hoboken). (2018) 70:1192–7. doi: 10.1002/acr.23461 29226569

[21] Gaitatzis A Carroll K Majeed A Sander JW. The epidemiology of the comorbidity of epilepsy in the general population. Epilepsia. (2004) 45:1613–22. doi: 10.1111/j.0013-9580.2004.17504.x 15571520

[22] Davies NM Holmes MV Davey Smith G. Reading Mendelian randomisation studies: a guide, glossary, and checklist for clinicians. Bmj. (2018) 362:k601. doi: 10.1136/bmj.k601 30002074 PMC6041728

[23] Hemani G Zheng J Elsworth B Wade KH Haberland V Baird D. The MR-Base platform supports systematic causal inference across the human phenome. Elife. (2018) 7:e34408. doi: 10.7554/eLife.34408 29846171 PMC5976434

[24] Skrivankova VW Richmond RC Woolf BAR Davies NM Swanson SA VanderWeele TJ. Strengthening the reporting of observational studies in epidemiology using mendelian randomisation (STROBE-MR): explanation and elaboration. Bmj. (2021) 375:n2233. doi: 10.1136/bmj.n2233 34702754 PMC8546498

[25] Okada Y Wu D Trynka G Raj T Terao C Ikari K. Genetics of rheumatoid arthritis contributes to biology and drug discovery. Nature. (2014) 506:376–81. doi: 10.1038/nature12873 PMC3944098 24390342

[26] Kurki MI Karjalainen J Palta P Sipilä TP Kristiansson K Donner K. FinnGen: Unique genetic insights from combining isolated population and national health register data. medRxiv. (2022). 2022.03.03.22271360. doi: 10.1101/2022.03.03.22271360

[27] Pritchard JK Przeworski M. Linkage disequilibrium in humans: models and data. Am J Hum Genet. (2001) 69:1–14. doi: 10.1086/321275 11410837 PMC1226024

[28] Pistis G Porcu E Vrieze SI Sidore C Steri M Danjou F. Rare variant genotype imputation with thousands of study-specific whole-genome sequences: implications for cost-effective study designs. Eur J Hum Genet. (2015) 23:975–83. doi: 10.1038/ejhg.2014.216 PMC4463504 25293720

[29] Johnson EL Krauss GL Lee AK Schneider ALC Dearborn JL Kucharska-Newton AM. Association between midlife risk factors and late-onset epilepsy: results from the atherosclerosis risk in communities study. JAMA Neurol. (2018) 75:1375–82. doi: 10.1001/jamaneurol.2018.1935 PMC6248112 30039175

[30] Zhang ZZ Wang MM Yuan S Liu XF. Alcohol, coffee, and milk intake in relation to epilepsy risk. Nutrients. (2022) 14(6):1153. doi: 10.3390/nu14061153 35334809 PMC8951548

[31] Larsson SC Burgess S. Appraising the causal role of smoking in multiple diseases: A systematic review and meta-analysis of Mendelian randomization studies. Ebiomedicine. (2022) 82:104154. doi: 10.1016/j.ebiom.2022.104154 35816897 PMC9278068

[32] Bowden J Del Greco MF Minelli C Davey Smith G Sheehan N Thompson J. A framework for the investigation of pleiotropy in two-sample summary data Mendelian randomization. Stat Med. (2017) 36:1783–802. doi: 10.1002/sim.7221 PMC5434863 28114746

[33] Bowden J Davey Smith G Burgess S. Mendelian randomization with invalid instruments: effect estimation and bias detection through Egger regression. Int J Epidemiol. (2015) 44:512–25. doi: 10.1093/ije/dyv080 PMC4469799 26050253

[34] Bowden J Davey Smith G Haycock PC Burgess S. Consistent estimation in mendelian randomization with some invalid instruments using a weighted median estimator. Genet Epidemiol. (2016) 40:304–14. doi: 10.1002/gepi.21965 PMC4849733 27061298

[35] Rana A Musto AE. The role of inflammation in the development of epilepsy. J Neuroinflammation. (2018) 15:144. doi: 10.1186/s12974-018-1192-7 29764485 PMC5952578

[36] Friedman A Dingledine R. Molecular cascades that mediate the influence of inflammation on epilepsy. Epilepsia. (2011) 52 Suppl 3:33–9. doi: 10.1111/j.1528-1167.2011.03034.x PMC3638118 21542844

[37] Ngugi AK Bottomley C Kleinschmidt I Wagner RG Kakooza-Mwesige A Ae-Ngibise K. Prevalence of active convulsive epilepsy in sub-Saharan Africa and associated risk factors: cross-sectional and case-control studies. Lancet Neurol. (2013) 12:253–63. doi: 10.1016/S1474-4422(13)70003-6 PMC3581814 23375964

[38] Uludag IF Duksal T Tiftikcioglu BI Zorlu Y Ozkaya F Kirkali G. IL-1β, IL-6 and IL1Ra levels in temporal lobe epilepsy. Seizure. (2015) 26:22–5. doi: 10.1016/j.seizure.2015.01.009 25799897

[39] Youn Y Sung IK Lee IG. The role of cytokines in seizures: interleukin (IL)-1β, IL-1Ra, IL-8, and IL-10. Korean J Pediatr. (2013) 56:271–4. doi: 10.3345/kjp.2013.56.7.271 PMC3728444 23908665

[40] Aronica E Crino PB. Inflammation in epilepsy: clinical observations. Epilepsia. (2011) 52 Suppl 3:26–32. doi: 10.1111/j.1528-1167.2011.03033.x 21542843

[41] Ravizza T Gagliardi B Noé F Boer K Aronica E Vezzani A. Innate and adaptive immunity during epileptogenesis and spontaneous seizures: evidence from experimental models and human temporal lobe epilepsy. Neurobiol Dis. (2008) 29:142–60. doi: 10.1016/j.nbd.2007.08.012 17931873

[42] Wallenstein MC. Attenuation of epileptogenesis by nonsteroidal anti-inflammatory drugs in the rat. Neuropharmacology. (1991) 30:657–63. doi: 10.1016/0028-3908(91)90087-R 1922684

[43] Hughes EG Peng X Gleichman AJ Lai M Zhou L Tsou R. Cellular and synaptic mechanisms of anti-NMDA receptor encephalitis. J Neurosci. (2010) 30:5866–75. doi: 10.1523/JNEUROSCI.0167-10.2010 PMC2868315 20427647

[44] Irani SR Bera K Waters P Zuliani L Maxwell S Zandi MS. N-methyl-D-aspartate antibody encephalitis: temporal progression of clinical and paraclinical observations in a predominantly non-paraneoplastic disorder of both sexes. Brain. (2010) 133:1655–67. doi: 10.1093/brain/awq113 PMC2877907 20511282

[45] Dalmau J Lancaster E Martinez-Hernandez E Rosenfeld MR Balice-Gordon R. Clinical experience and laboratory investigations in patients with anti-NMDAR encephalitis. Lancet Neurol. (2011) 10:63–74. doi: 10.1016/S1474-4422(10)70253-2 21163445 PMC3158385

[46] Xie YF Huo FQ Tang JS. Cerebral cortex modulation of pain. Acta Pharmacol Sin. (2009) 30:31–41. doi: 10.1038/aps.2008.14 19079295 PMC4006538

[47] Sandström A Ellerbrock I Jensen KB Martinsen S Altawil R Hakeberg P. Altered cerebral pain processing of noxious stimuli from inflamed joints in rheumatoid arthritis: An event-related fMRI study. Brain Behav Immun. (2019) 81:272–9. doi: 10.1016/j.bbi.2019.06.024 31228612

[48] Flodin P Martinsen S Altawil R Waldheim E Lampa J Kosek E. Intrinsic brain connectivity in chronic pain: A resting-state fMRI study in patients with rheumatoid arthritis. Front Hum Neurosci. (2016) 10:107. doi: 10.3389/fnhum.2016.00107 27014038 PMC4791375

[49] Jones AK Derbyshire SW. Reduced cortical responses to noxious heat in patients with rheumatoid arthritis. Ann Rheum Dis. (1997) 56:601–7. doi: 10.1136/ard.56.10.601 PMC1752267 9389221

[50] Bourgeois P Rivest J Bocti C. Rheumatoid meningitis presenting with stroke-like episodes. Neurology. (2014) 82:1564–5. doi: 10.1212/WNL.0000000000000366 PMC4011466 24647026

[51] Kurne A Karabudak R Karadag O Yalcin-Cakmakli G Karli-Oguz K Yavuz K. An unusual central nervous system involvement in rheumatoid arthritis: combination of pachymeningitis and cerebral vasculitis. Rheumatol Int. (2009) 29:1349–53. doi: 10.1007/s00296-008-0810-6 19093117

[52] Guadalupe Loya-de la Cerda D Avilés-Solís JC Delgado-Montemayor MJ Camara-Lemarroy CR Galarza-Delgado D. Isolated rheumatoid arthritis-associated cerebral vasculitis: a diagnostic challenge. Joint Bone Spine. (2013) 80:88–90. doi: 10.1016/j.jbspin.2012.06.014 22858148

[53] Khan O Aslam S Mohammadrezaei F Wilches RDM Mehrabi J Yehounatan M. New-onset seizures: an unusual neurologic manifestation of rheumatoid arthritis. Oxf Med Case Rep. (2024) 2024:omad159. doi: 10.1093/omcr/omad159 PMC10873692 38370505

[54] Roberts AJ Keith LD. Corticosteroids enhance convulsion susceptibility via central mineralocorticoid receptors. Psychoneuroendocrinology. (1995) 20:891–902. doi: 10.1016/0306-4530(95)00016-X 8834095

[55] Thomas E Leroux JL Hellier JP Blotman F. Seizure and methotrexate therapy in rheumatoid arthritis. J Rheumatol. (1993) 20:1632. doi: 10.1093/omcr/omad159 8164234

[56] Hill ME Gordon C Situnayake RD Heath DA. Sulfasalazine induced seizures and dysphasia. J Rheumatol. (1994) 21:748–9. doi: 10.1016/0306-4530(95)00016-x 7913503

[57] Laxer KD Trinka E Hirsch LJ Cendes F Langfitt J Delanty N. The consequences of refractory epilepsy and its treatment. Epilepsy Behav. (2014) 37:59–70. doi: 10.1016/j.yebeh.2014.05.031 24980390

[58] de Tisi J Bell GS Peacock JL McEvoy AW Harkness WF Sander JW. The long-term outcome of adult epilepsy surgery, patterns of seizure remission, and relapse: a cohort study. Lancet. (2011) 378:1388–95. doi: 10.1016/S0140-6736(11)60890-8 22000136

[59] Bowden J Hemani G Davey Smith G. Invited commentary: detecting individual and global horizontal pleiotropy in mendelian randomization-A job for the humble heterogeneity statistic? Am J Epidemiol. (2018) 187:2681–5. doi: 10.1093/aje/kwy185 PMC6269239 30188969

[60] Verbanck M Chen CY Neale B Do R. Detection of widespread horizontal pleiotropy in causal relationships inferred from Mendelian randomization between complex traits and diseases. Nat Genet. (2018) 50:693–8. doi: 10.1038/s41588-018-0099-7 PMC6083837 29686387

