# Supplementary material for: Causal relationship between rheumatoid arthritis and epilepsy in a European population: a univariate and multivariate Mendelian randomization study
Source: Front Immunol. 2024 May 16;15:1389549. doi: 10.3389/fimmu.2024.1389549 (PMC11137193; doi:10.3389/fimmu.2024.1389549)
Supplement: Supplementary file 2 [file Table_2.pdf]

STROBE-MR checklist of recommended items to address in reports of Mendelian randomization studies[1 2]

Item No.	Section	Checklist item	Page No.	Relevant text from manuscript
1	**TITLE and ABSTRACT**	Indicate Mendelian randomization (MR) as the study's design in the title and/or the abstract if that is a main purpose of the study	1-2	Causal relationship between rheumatoid arthritis and epilepsy: a univariate and multivariate Mendelian randomization study<br>The aim of this study was to explore the causal relationship between RA and epilepsy in a European population using Mendelian randomization (MR).
	**INTRODUCTION**			
2	**Background**	Explain the scientific background and rationale for the reported study. What is the exposure? Is a potential causal relationship between exposure and outcome plausible? Justify why MR is a helpful method to address the study question	2-3	Epilepsy is a prevalent and highly disabling chronic central nervous system disorder characterized by sudden abnormal discharges of neurons in the brain resulting in transient dysfunction of the brain.<br>In recent years, a number of studies have reported an association between RA and epilepsy.<br>Genetic variants are identified at conception through random assignment, which means that they are not affected by other factors such as behavioral, environmental, and social factors, allowing MR to avoid confounding by confounding factors and reverse causation to the greatest extent possible.
3	**Objectives**	State specific objectives clearly, including pre-specified causal hypotheses (if any). State that MR is a method that, under specific assumptions, intends to estimate causal effects	3	Mendelian randomization (MR) is an innovative epidemiological approach that uses genetic variants such as single nucleotide diversity (SNPs) as instrumental variables (IVs) to infer potential causal relationships between exposures and outcomes.
	**METHODS**			
4	**Study design and data sources**	Present key elements of the study design early in the article. Consider including a table listing sources of data for all phases of the study. For each data source contributing to the analysis, describe the following:		

		a)	Setting: Describe the study design and the underlying population, if possible. Describe the setting, locations, and relevant dates, including periods of recruitment, exposure, follow-up, and data collection, when available.	4,Table S1	European population.Table S1.
		b)	Participants: Give the eligibility criteria, and the sources and methods of selection of participants. Report the sample size, and whether any power or sample size calculations were carried out prior to the main analysis	4	The GWAS summary data on RA (GWAS ID: ebi-a-GCST90013534) includes 58,284 individuals of both sexes, including 14,361 cases and 43,923 controls, with 13,108,512 SNPs. The GWAS summary data for epilepsy (GWAS ID: finn-b-G6_EPLEPSY) includes 182,367 individuals of both sexes, including 6,260 cases and 176,107 controls, with 16,380,349 SNPs.
		c)	Describe measurement, quality control and selection of genetic variants	N/A	N/A
		d)	For each exposure, outcome, and other relevant variables, describe methods of assessment and diagnostic criteria for diseases	N/A	N/A
		e)	Provide details of ethics committee approval and participant informed consent, if relevant	4	GWAS summary data on RA and epilepsy were extracted from the IEU Open GWAS project (https://gwas.mrcieu.ac.uk/), and the data are publicly released and available, so no additional ethical approval was required.
5	**Assumptions**		Explicitly state the three core IV assumptions for the main analysis (relevance, independence and exclusion restriction) as well assumptions for any additional or sensitivity analysis	3	To ensure the reliability of the results of the MR analyses, the selected IVs had to fulfill three key assumptions: (1) there is a strong association between the IVs and RA; (2) the IVs are not associated with any other potential confounders that may affect RA and epilepsy; and (3) the IVs affect epilepsy only through RA.
6	**Statistical methods: main analysis**		Describe statistical methods and statistics used		
		a)	Describe how quantitative variables were handled in the analyses (i.e., scale, units, model)	N/A	N/A
		b)	Describe how genetic variants were handled in the analyses and, if applicable, how their weights were selected	4	(1) To ensure that SNPs were significantly associated with our exposure factors of interest, SNPs with P < 5 × 10-8 were selected; (2) to avoid the influence of linkage disequilibrium (LD) among selected SNPs, we referred to the European population samples and used a thresholds such as r2 < 0.001 and distance = 10000 kb for LD-clumping to exclude SNPs with strong LD.

					F-statistic was incorporated to ensure that the selected IVs were strong IVs, and usually variables with F-statistic > 10 were defined as strong IVs.
		c)	Describe the MR estimator (e.g. two-stage least squares, Wald ratio) and related statistics. Detail the included covariates and, in case of two-sample MR, whether the same covariate set was used for adjustment in the two samples	4	UVMR was first used to investigate whether there is a causal relationship between RA and epilepsy. Then, we used MVMR to further verify the reliability of the previously derived causal relationship after adjusting for hypertension (27), alcohol consumption (28), and smoking (29), three common risk factors for epilepsy reported in previous studies.
		d)	Explain how missing data were addressed	N/A	N/A
		e)	If applicable, indicate how multiple testing was addressed	N/A	N/A
7	**Assessment of assumptions**		Describe any methods or prior knowledge used to assess the assumptions or justify their validity	4-5	Inverse Variance Weight (IVW), MR-Egger, and Weighted Median (WM) analyses were used in our study. They make different assumptions about possible horizontal pleiotropy: IVW assumes that all IVs are free of horizontal pleiotropy (30); MR-Egger assumes that all IVs are horizontally polytropic (31); and WM assumes that horizontal pleiotropy can exist in 50% of the IVs (32). Of these, IVW served as our primary analytic method because it allows for the most accurate causal assessment in the absence of horizontal pleiotropy (30). If horizontal pleiotropy occurs between SNPs, the results obtained by the two methods MR-Egger and WM can be referred to.
8	**Sensitivity analyses and additional analyses**		Describe any sensitivity analyses or additional analyses performed (e.g. comparison of effect estimates from different approaches, independent replication, bias analytic techniques, validation of instruments, simulations)	5	The significance of sensitivity analysis is to ensure the robustness of MR analysis results, which mainly include heterogeneity analysis, horizontal pleiotropy analysis, and the leave-one-out test
9	**Software and pre-registration**				
		a)	Name statistical software and package(s), including version and settings used	4	We performed all MR as well as correlation analyses in R (4.3.2) software using the three R packages: TwoSample MR, MR-PRESSOR, and Mendelian Randomization.
		b)	State whether the study protocol and details were pre-registered (as well as when and where)	N/A	N/A

	**RESULTS**				
10	**Descriptive data**				
		a)	Report the numbers of individuals at each stage of included studies and reasons for exclusion. Consider use of a flow diagram	Figure 1	Figure 1
		b)	Report summary statistics for phenotypic exposure(s), outcome(s), and other relevant variables (e.g. means, SDs, proportions)	Table S2	Table S2
		c)	If the data sources include meta-analyses of previous studies, provide the assessments of heterogeneity across these studies	N/A	N/A
		d)	For two-sample MR:<br>i. Provide justification of the similarity of the genetic variant-exposure associations between the exposure and outcome samples<br>ii. Provide information on the number of individuals who overlap between the exposure and outcome studies	5	After rigorous screening of IVs according to previously developed criteria, a total of 90 SNPs met the requirements to be included in this study, of which rs34536443 was excluded from MR analysis because it was found to have a palindrome with an intermediate allele frequency. All SNPs were strong instrumental variables (F-statistic > 10). Details
11	**Main results**				
		a)	Report the associations between genetic variant and exposure, and between genetic variant and outcome, preferably on an interpretable scale	5	IVW method showed a positive association between RA and epilepsy risk.
		b)	Report MR estimates of the relationship between exposure and outcome, and the measures of uncertainty from the MR analysis, on an interpretable scale, such as odds ratio or relative risk per SD difference	5	(OR=1.038,95% CI=1.007-1.038, p=0.017); MR-Egger: OR=1.045,95% CI=0.998-1.095, p=0.066; Weighted median: OR=0.997,95%CI=0.949-1.047, p=0.897.
		c)	If relevant, consider translating estimates of relative risk into absolute risk for a meaningful time period	N/A	N/A
		d)	Consider plots to visualize results (e.g. forest plot, scatterplot of associations between genetic variants and outcome versus between genetic variants and exposure)	Figure 2-3	Figure 2-3
12	**Assessment of assumptions**				
		a)	Report the assessment of the validity of the assumptions	6	Considering that the results of UVMR may be affected by potential confounders, we performed further MVMR analysis. After coordinating

					hypertension, alcohol consumption, and smoking, the IVW method showed that there was still a positive causal association between RA and epilepsy risk.
		b)	Report any additional statistics (e.g., assessments of heterogeneity across genetic variants, such as I^2, Q statistic or E-value)	6,Table S3	(OR=1.049,95% CI=1.011-1.087, p=0.010).<br>Table S3
13	**Sensitivity analyses and additional analyses**				
		a)	Report any sensitivity analyses to assess the robustness of the main results to violations of the assumptions	5-6	The results of sensitivity analyses supported a causal relationship between genetically predicted RA and epilepsy. p-value > 0.05 in Cochran Q statistics indicated the absence of heterogeneity. A p-value > 0.05 in the MR-Egger intercept test indicated the absence of horizontal pleiotropy. leave-one-out test indicated that the overall findings were not influenced by a single SNP. Due to the absence of heterogeneity as well as horizontal pleiotropy, we consider the results obtained by the IVW method to be reliable. The results of the analysis of Cochran Q statistics, MR-Egger intercept can be seen in Tables S3-S4. Figures 2-4 show scatter plots, funnel plots, and leave-one-out test plots of UVMR results regarding the relationship between RA and epilepsy, respectively.
		b)	Report results from other sensitivity analyses or additional analyses	5-6	p-value > 0.05 in Cochran Q statistics indicated the absence of heterogeneity. A p-value > 0.05 in the MR-Egger intercept test indicated the absence of horizontal pleiotropy. leave-one-out test indicated that the overall findings were not influenced by a single SNP.
		c)	Report any assessment of direction of causal relationship (e.g., bidirectional MR)	N/A	N/A
		d)	When relevant, report and compare with estimates from non-MR analyses	N/A	N/A
		e)	Consider additional plots to visualize results (e.g., leave-one-out analyses)	Figure 4-5	Figure 4-5
	**DISCUSSION**				

14	**Key results**	Summarize key results with reference to study objectives	6	The present study assessed the causal relationship existing between RA and epilepsy by MR analysis. The results of our UVMR analysis suggest that RA leads to an increased risk of epilepsy, which was similarly confirmed by the results of further MVMR analysis. Our findings provide important clues to further understand the mechanisms underlying this association and emphasize the importance of timely monitoring and prevention of epilepsy in patients diagnosed with RA.
15	**Limitations**	Discuss limitations of the study, taking into account the validity of the IV assumptions, other sources of potential bias, and imprecision. Discuss both direction and magnitude of any potential bias and any efforts to address them	8	At the same time, we must also recognize some shortcomings. First, the samples included in this study were of European origin and therefore may not be generalizable to other populations. Second, due to data limitations we were unable to stratify by gender and age. GWAS data with more details are needed in the future to more fully assess the differences between the study subjects.
16	**Interpretation**			
	a)	Meaning: Give a cautious overall interpretation of results in the context of their limitations and in comparison with other studies	7	To the best of our knowledge, this study is the first to validate the causal relationship between RA and epilepsy using an innovative method such as MR, which can minimize confounding by confounding factors and reverse causality and can be performed using existing publicly available and reliable data, which is more cost-effective and feasible than other studies. In addition, our data sources are recent GWAS data with large sample sizes, and we have conducted a series of sensitivity analyses to ensure the credibility and robustness of our results.
	b)	Mechanism: Discuss underlying biological mechanisms that could drive a potential causal relationship between the investigated exposure and the outcome, and whether the gene-environment equivalence assumption is reasonable. Use causal language carefully, clarifying that IV estimates may provide causal effects only under certain assumptions	7	Several aspects may be involved, including an inflammatory response, autoimmunity, and painful stimuli.
	c)	Clinical relevance: Discuss whether the results have clinical or public policy relevance, and to what extent they inform effect sizes of possible interventions	8	Based on this finding, we suggest that monitoring of epilepsy risk in patients diagnosed with RA as well as individualized assessment should be strengthened in clinical practice, and further

				studies are needed in the future to explore the potential mechanism of action between the two.
17	**Generalizability**	Discuss the generalizability of the study results (a) to other populations, (b) across other exposure periods/timings, and (c) across other levels of exposure	8	the samples included in this study were of European origin and therefore may not be generalizable to other populations
	**OTHER INFORMATION**			
18	**Funding**	Describe sources of funding and the role of funders in the present study and, if applicable, sources of funding for the databases and original study or studies on which the present study is based	N/A	The submission page provides a description.
19	**Data and data sharing**	Provide the data used to perform all analyses or report where and how the data can be accessed, and reference these sources in the article. Provide the statistical code needed to reproduce the results in the article, or report whether the code is publicly accessible and if so, where	8	All of the GWAS summary data in our study is available at the GWAS Catalog(https://gwas.mrcieu.ac.uk/).
20	**Conflicts of Interest**	All authors should declare all potential conflicts of interest	8	The authors declare no conflict of interest.

This checklist is copyrighted by the Equator Network under the Creative Commons Attribution 3.0 Unported (CC BY 3.0) license.

1. Skrivankova VW, Richmond RC, Woolf BAR, Yarmolinsky J, Davies NM, Swanson SA, et al. Strengthening the Reporting of Observational Studies in Epidemiology using Mendelian Randomization (STROBE-MR) Statement. JAMA. 2021;under review.

2. Skrivankova VW, Richmond RC, Woolf BAR, Davies NM, Swanson SA, VanderWeele TJ, et al. Strengthening the Reporting of Observational Studies in Epidemiology using Mendelian Randomisation (STROBE-MR): Explanation and Elaboration. BMJ. 2021;375:n2233.
